# QUALITY OF LIFE AND NUTRITION SCREENING: OVERLAPS, OPPORTUNITIES, AND IMPLICATIONS FOR HEALTHY AGING

**DOI:** 10.1093/geroni/igad104.3544

**Published:** 2023-12-21

**Authors:** Mary Beth Arensberg, Jaime Gahche, Raquel Clapés Pemau, Kirk Kerr, Johanna Dwyer

**Affiliations:** Abbott Nutrition, Columbus, Ohio, United States; National Institutes of Health, Bethesda, Maryland, United States; Abbott, Granada, Andalucia, Spain; Abbott Nutrition, Columbus, Ohio, United States; National Institutes of Health, Bethesda, Maryland, United States

## Abstract

Quality of life (QoL) is important for healthy aging, both for older adults themselves and healthcare providers/systems caring for them. Eating/nutritious food are essential for maintaining good nutrition status, provide pleasure, help older adults remain healthy/independent, and contribute to important QoL domains. Yet research on nutrition and QoL is limited. This study describes connections between QoL and nutrition by examining QoL instruments and nutrition screening tools used with community-living older adults. 20 validated QoL instruments were evaluated to determine if they included nutrition-related items involving 8 previously identified QoL domains. 75% of QoL screening tools included at least 1 nutrition-related item, most commonly in autonomy (n=11), physical health (n=7), social connection (n=3), environment (n=3), emotional state (n=2), mental health (n=2), and personhood (n=1) domains; none in spiritual feeling domain. 16 validated nutrition screening tools were evaluated to determine how their nutrition items related to the same specific QoL domains; all tools included at least 1 nutrition item related to physical health (n=16). Other domains represented by nutrition items were autonomy (n= 9), emotional state (n=5), social connection (n=5), environment (n=4), and mental health (n=3). Although commonalities existed between QoL instruments and nutrition screening tools in types of nutrition-related items included, there were many inconsistencies/gaps. Only 1 QoL instrument and no nutrition screening tools included items in the personhood domain that were related to enjoyment in eating. Nutrition is a potentially modifiable factor benefiting healthy aging and QoL outcomes; these gaps represent opportunities for further research and policy and program development.

